# Zoonotic *Streptococcus canis* Bacteremia Following a Dog Scratch in an Elderly Patient With a Nonconditional Pacemaker

**DOI:** 10.1155/crdi/2485747

**Published:** 2026-02-27

**Authors:** Kamel Alachraf, Patrick Trouten, Jessica Thayer

**Affiliations:** ^1^ Department of Medicine, WVU Health, Morgantown, West Virginia, USA

**Keywords:** bacteremia, cardiac implantable electronic device, dog-associated infection, *Streptococcus canis*, zoonotic infection

## Abstract

*Streptococcus canis* is a β‐hemolytic Group G streptococcus commonly found in the microbiome of dogs and cats and is an uncommon cause of invasive human infection. Although typically regarded as a veterinary pathogen, *S. canis* has been reported to cause bacteremia, endocarditis, and other severe infections in humans, particularly in older adults with significant comorbidities or implanted medical devices. We describe a case of *Streptococcus canis* bacteremia in an 89‐year‐old woman with multiple comorbidities and a nonconditional permanent pacemaker who presented with fever, dyspnea, and severe lower back pain. Blood cultures grew *S. canis*, identified using standard microbiologic techniques. The clinical course raised concern for metastatic infection and pacemaker involvement. Imaging of the thoracic and lumbar spine demonstrated no evidence of discitis or osteomyelitis, and both transthoracic and transesophageal echocardiography showed no valvular or pacemaker lead vegetations. Further history revealed a dog scratch several weeks prior to presentation that resulted in skin disruption, representing a plausible portal of entry. The patient was treated with intravenous ceftriaxone with rapid clinical improvement, resolution of hypoxia, clearance of bacteremia, and declining inflammatory markers. She was discharged to inpatient rehabilitation to complete a 2‐week course of antimicrobial therapy and recovered without evidence of recurrent infection. This case underscores *Streptococcus canis* as an uncommon but clinically relevant zoonotic pathogen and highlights the importance of detailed exposure history, appropriate microbiologic identification, and careful evaluation for device‐related infection in high‐risk patients.

## 1. Background

*S. canis* is the most frequently isolated streptococcus from dogs and cats. It is a β‐hemolytic, Group G (pyogenic) streptococcus that colonizes the skin, genital, and gastrointestinal tracts of healthy dogs and cats [1]. While *S. canis* is primarily considered a veterinary pathogen, it can occasionally cause invasive infections in humans, particularly in immunocompromised individuals or those with significant comorbidities or implanted medical devices. Reported human infections include bacteremia, cellulitis, necrotizing fasciitis, septic arthritis, and endocarditis, often in the context of zoonotic transmission [2].

## 2. Clinical Course

An 89‐year‐old female presented to the Emergency Department with a 4‐day history of fever, chills, shortness of breath, and general malaise, all of which had worsened over the preceding 24 h. She also reported lower back pain radiating to both legs. Her past medical history was significant for chronic kidney disease Stage IV, heart failure with preserved ejection fraction (HFpEF), atrial fibrillation, and a permanent pacemaker.

The patient’s initial vital signs were notable for a temperature of 38.6°C, blood pressure of 114/32 mmHg, heart rate of 70 bpm, respiratory rate of 19, and oxygen saturation of 94% on room air. The patient appeared ill and in distress, primarily due to right lower extremity and lower back pain. Integumentary exam revealed scattered bruising on the upper extremities, without signs of cellulitis or stigmata of endocarditis. Cardiac, pulmonary, abdominal, and neurologic examinations were unremarkable. However, the patient developed a new oxygen requirement of 2 L/min via nasal cannula the following morning.

Initial laboratory evaluation demonstrated anemia, acute kidney injury on chronic kidney disease, elevated transaminases, and a markedly elevated C‐reactive protein (Tables 1 and 2). Computed tomography of the chest, abdomen, and pelvis demonstrated subtle left lower lobe ground‐glass opacities. Urinalysis showed pyuria, though urine cultures were negative (Tables 1 and 2).

**TABLE 1:** Patient’s laboratory values on admission.

Hemoglobin	10.6 g/dL	Low
MCV	92.7 fL	Normal
WBC	9.4 × 10^3^/μL	Normal
Neutrophils	6.59 × 10^3^/μL	Normal
Sodium	138 mmol/L	Normal
Potassium	4.7 mmol/L	Normal
Chloride	107 mmol/L	Normal
Bicarbonate	26 mmol/L	Normal
Calcium	8.5 mg/dL	Normal
Magnesium	2.4 mg/dL	Normal
Bilirubin	1.1 mg/dL	Normal
Glucose	149 mg/dL	Elevated
Creatinine	1.94 mg/dL	Elevated
AST	53 U/L	Elevated
ALT	42 U/L	Elevated
ALP	74 U/L	Normal
Albumin	3.4 g/dL	Normal
CRP	118.5 mg/L	Elevated

**TABLE 2:** Abbreviations.

AKI	Acute kidney injury
ALT	Alanine aminotransferase
ALP	Alkaline phosphatase
AST	Aspartate aminotransferase
BMP	Basic metabolic panel
CBC	Complete blood count
CKD	Chronic kidney disease
CRP	C‐reactive protein
CT	Computed tomography
EF	Ejection fraction
HFpEF	Heart failure with preserved ejection fraction
IV	Intravenous
LFTs	Liver function tests
MRI	Magnetic resonance imaging
OPAT	Outpatient parenteral antimicrobial therapy
SpO_2_	Peripheral oxygen saturation
TEE	Transesophageal echocardiogram
TTE	Transthoracic echocardiogram
UA	Urinalysis
WBC	White blood cell count

Empiric antimicrobial therapy with doxycycline and ceftriaxone was initiated. Urinary antigen testing for *Legionella pneumophila* and *Streptococcus pneumoniae* was obtained as part of the standard diagnostic evaluation for unexplained hypoxia. Blood cultures subsequently returned positive for Gram‐positive cocci, prompting substitution of doxycycline with vancomycin. The organism was later identified as *Streptococcus canis* using matrix‐assisted laser desorption/ionization time‐of‐flight mass spectrometry (MALDI‐TOF MS). Antimicrobial susceptibility testing was performed using standard laboratory methods and demonstrated susceptibility to beta‐lactam antibiotics, including ceftriaxone, which guided definitive therapy. On further questioning, the patient reported a dog scratch from her daughter’s pet several weeks prior to admission that resulted in a break in the skin with minor bleeding but did not require medical attention. She denied other animal exposures or recent open wounds.

**FIGURE 1:**
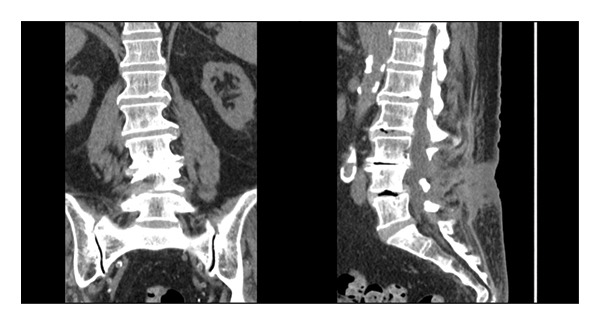
Computed tomography (CT) of the lumbar spine without intravenous contrast, sagittal and coronal reconstructions, demonstrating no prevertebral or paraspinal soft tissue edema to suggest discitis, osteomyelitis, or septic arthritis.

**FIGURE 2:**
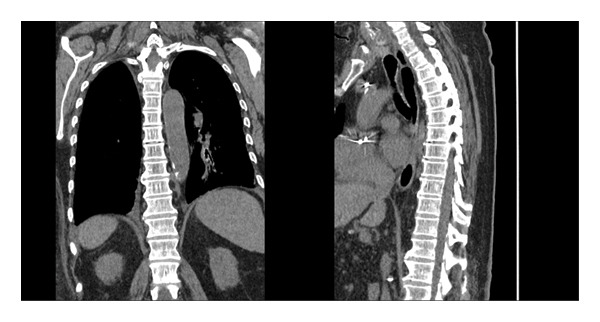
Computed tomography (CT) of the thoracic spine without intravenous contrast and sagittal and coronal reconstructions, demonstrating no evidence of osseous involvement.

**FIGURE 3:**
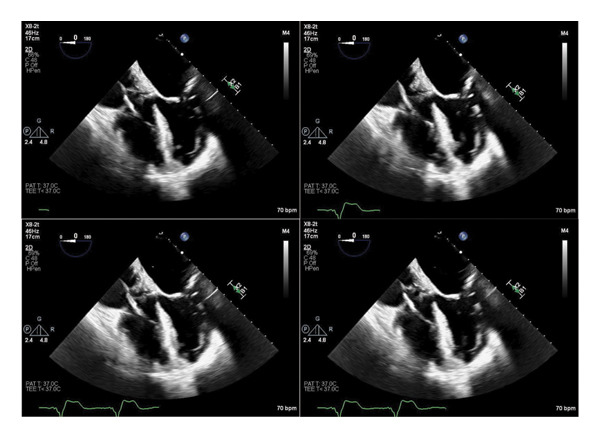
Transesophageal echocardiogram (TEE), midesophageal four‐chamber view, demonstrating no evidence of valvular vegetations or pacemaker lead–associated vegetations.

After 2 days of targeted therapy with ceftriaxone, the patient showed significant clinical improvement, with resolution of her oxygen requirement and decreasing inflammatory markers. However, she continued to experience severe lumbar back pain. A CT of the thoracic and lumbar spine findings was interpreted as degenerative and not consistent with infectious involvement (Figures 1 and 2). MRI was not feasible due to the patient’s non conditional pacemaker. Given her bacteremia and implanted cardiac device, a transthoracic echocardiogram (TTE) was performed and was unrevealing. A follow‐up transesophageal echocardiogram (TEE), done per infectious disease recommendations, showed no valvular or lead vegetations (Figure 3).

The patient demonstrated rapid clinical improvement with resolution of hypoxia, clearance of bacteremia, and declining inflammatory markers. Repeat blood cultures were negative. She was discharged to inpatient rehabilitation to complete a 2‐week course of intravenous ceftriaxone, after which she was able to return home and recovered without recurrence of infection.

## 3. Discussion

This case illustrates *S. canis* as a rare but significant cause of bacteremia in humans, particularly in elderly or immunocompromised hosts. Although typically associated with canine and feline microbiome, *S. canis* can be transmitted zoonotically, even from seemingly benign exposures. Although most human *S. canis* infections are linked to animal exposure with an apparent bite or scratch, several well documented cases include septicemia in a dog owner without any bite and native mitral valve endocarditis after a simple dog contact without any trauma [3–5]. In this case, the only identified risk factor was a minor dog scratch several weeks prior to symptom onset.

The patient presented with sepsis and developed a new oxygen requirement, suggesting pulmonary involvement, although imaging showed only minimal, nonspecific ground‐glass changes. Although the patient’s clinical status rapidly improved with antibiotics, previously reported cases of sepsis or valvular involvement prompted early infectious disease consultation and extensive imaging for sources of colonization. The patient’s severe lumbar back pain initially raised concern for osseous involvement. Moreover, her permanent pacemaker raised concern for device‐related infection, prompting cardiac imaging via TTE then TEE.

Although *S. canis* is rarely encountered in human infections, literature reports demonstrate its potential for causing invasive disease, including endocarditis, septic arthritis, and necrotizing infections. Causes often arise without obvious bite or scratch injuries, making clinical suspicion difficult [6–9]. Risk factors for severe disease include advanced age, underlying comorbidities, immunosuppression, and the presence of prosthetic material. Identification and susceptibility testing are essential to guide therapy, as *S. canis* is typically susceptible to beta‐lactam antibiotics. Delayed recognition and treatment may result in serious complications, including valve destruction, embolic events, and increased mortality; notably, Group C and Group G streptococcal endocarditis of which *Streptococcus canis* is a member has been associated with mortality rates approaching 17% [10].

## 4. Conclusion

This case highlights the importance of considering zoonotic pathogens in patients with unclear sources of infection and animal exposure. Even if initial clinical suspicion for zoonotic infection is low, thorough history of potential infectious exposures can guide infectious evaluation, particularly when the initial infectious source is unclear. Without advanced microbiology laboratory testing to isolate rare species, history of zoonotic or pet exposure would have been the only evidence of infectious source, given her negative urinary and respiratory cultures. Prompt diagnosis, appropriate imaging, and tailored antimicrobial therapy led to a favorable outcome in this complex patient with multiple comorbidities and an indwelling cardiac device.

Abbreviations used throughout the manuscript are summarized in Table 2.

## Funding

This work did not receive any specific grant from funding agencies in the public, commercial, or not‐for‐profit sectors.

## Disclosure

The content is solely the responsibility of the authors and does not necessarily represent the official views of the National Institutes of Health.

## Consent

Consent was obtained from the patient prior to the use of de‐identified patient information for the purpose of writing and publishing this report. The West Virginia University Institutional Review Board reviewed the project and determined it was Not Human Subjects Research and that an IRB protocol was not required.

## Conflicts of Interest

The authors declare no conflicts of interest.

## Data Availability

No additional data are available beyond what are included in the manuscript. The clinical information presented has been de‐identified to protect patient confidentiality.
